# Ischemic Stroke, Glucocorticoids, and Remote Hippocampal Damage: A Translational Outlook and Implications for Modeling

**DOI:** 10.3389/fnins.2021.781964

**Published:** 2021-12-09

**Authors:** Natalia V. Gulyaeva, Mikhail V. Onufriev, Yulia V. Moiseeva

**Affiliations:** ^1^Laboratory of Functional Biochemistry of Nervous System, Institute of Higher Nervous Activity and Neurophysiology, Russian Academy of Sciences, Moscow, Russia; ^2^Research and Clinical Center for Neuropsychiatry of Moscow Healthcare Department, Moscow, Russia

**Keywords:** stroke, hippocampus, distant damage, cortisol, corticosterone, hypothalamic-pituitary-adrenal axis, glucocorticoids, neuroinflammation

## Abstract

Progress in treating ischemic stroke (IS) and its delayed consequences has been frustratingly slow due to the insufficient knowledge on the mechanism. One important factor, the hypothalamic-pituitary-adrenocortical (HPA) axis is mostly neglected despite the fact that both clinical data and the results from rodent models of IS show that glucocorticoids, the hormones of this stress axis, are involved in IS-induced brain dysfunction. Though increased cortisol in IS is regarded as a biomarker of higher mortality and worse recovery prognosis, the detailed mechanisms of HPA axis dysfunction involvement in delayed post-stroke cognitive and emotional disorders remain obscure. In this review, we analyze IS-induced HPA axis alterations and supposed association of corticoid-dependent distant hippocampal damage to post-stroke brain disorders. A translationally important growing point in bridging the gap between IS pathogenesis and clinic is to investigate the involvement of the HPA axis disturbances and related hippocampal dysfunction at different stages of SI. Valid models that reproduce the state of the HPA axis in clinical cases of IS are needed, and this should be considered when planning pre-clinical research. In clinical studies of IS, it is useful to reinforce diagnostic and prognostic potential of cortisol and other HPA axis hormones. Finally, it is important to reveal IS patients with permanently disturbed HPA axis. Patients-at-risk with high cortisol prone to delayed remote hippocampal damage should be monitored since hippocampal dysfunction may be the basis for development of post-stroke cognitive and emotional disturbances, as well as epilepsy.

## Introduction

…all models are approximations. Essentially, all models are wrong, but some are useful. However, the approximate nature of the model must always be borne in mind…

*George E.P. Box, Norman R. Draper, Response Surfaces, Mixtures, and Ridge Analyses (2007), 414.*

Ischemic stroke (IS) accounting for approximately 87% of stroke cases (Virani et al., 2021) is a severe neurological pathology with high mortality and frequent post-stroke delayed sequelae comprising both neurological and cognitive disorders. IS, a highly intricate and devastating neurological disease, is still among leading causes of death and disability worldwide. The mechanisms of IS pathogenesis are numerous and multifaceted, many essential details remain obscure and this may be the main reason why the therapy, though aimed at known key pathogenetic links, is not effective enough. The pathophysiology of IS includes sequential energy pumps failure and complex signaling cascades eventually resulting in neuronal cell death. Ischemic cascades involve hyperglutamatergic transmission associated with excessive glutamate release, increased intracellular sodium and calcium influx accompanied by free radical generation, secretion of inflammatory cytokines, activation of apoptotic and other pathways ultimately resulting in cell edema and demise. The drop in blood flow of a brain region during IS results in formation of a severe damaged ischemic area, the ischemic core, surrounded by the penumbra, a functionally damaged but potentially salvageable tissue. For several decades, the penumbra remains the main target for neuroprotective strategies, though remote brain regions are injured as well.

Although the treatment has improved in recent years, stroke is still the main reason for long-term disability (Feigin et al., 2019). Progress in treating IS and its delayed consequences has been frustratingly slow. For quite a few last decades, lots of candidate neuroprotective drugs have shown promising results in studies using rodent IS models and thousands papers are available showing positive results in pre-clinical IS studies. Yet, in clinical trials, these drugs have failed to show convincing results in efficacy and safety and none of them have shown beneficial effects (Dhir et al., 2020; Levard et al., 2021; Lyden, 2021). This disappointment is certainly related to an incomplete current picture of IS pathophysiology. Lack of valid and powerful animal models for human IS is regarded as a major reason for the failure to develop effective neuroprotective drugs (Narayan et al., 2021; Singh et al., 2021). Many authors point at this translational failure in IS treatment and note the urgent need to reorganize the design of stroke experimental setups. This may include use of pre-clinical models more accurately mimicking the clinical conditions of IS patients, including aged animals and IS models with comorbidities such as hypertension, diabetes, obesity and hyperlipidemia, as well as performing extensive pre-clinical stroke trials with continuous follow-up studies (Candelario-Jalil and Paul, 2020; Levard et al., 2021; Mizuma and Yenari, 2021).

The effects of IS on neurohumoral system underlying brain integrative control mechanisms are still neglected in IS models. Yet, stress is one of the major players in IS pathogenesis, and the stress response of a patient is relevant for the onset, course and outcome of IS. Stress-associated activation and dysregulation of HPA axis is accompanied by the release of excessive quantities of glucocorticoids (GCs) which induce signal transduction through specific receptors. Hippocampus, a brain region controlling both cognitive functions and emotions, is selectively vulnerable to stress and corticosteroid receptors are enriched in this limbic structure. Stress-induced glucocorticoid-dependent hippocampal damage is believed to be mediated by abnormal signaling through these receptors suggesting an association between hippocampal dysfunction and cognitive and emotional consequences of IS (see Gulyaeva, 2019a,b for review).

In this review, we analyze available data on the involvement of dysfunction of the hypothalamic-pituitary-adrenal (HPA) system in IS pathogenesis and putative association of corticoid-dependent distant hippocampal damage to post-stroke brain disorders. As a rule, this mechanism is not considered in animal IS studies. We discuss the necessity to take into account HPA axis malfunction in routine IS models used in pre-clinical studies.

## Ischemic Stroke and Glucocorticoids: Clinical and Pre-Clinical Data

Every stress leaves an indelible scar, and the organism pays for its survival after a stressful situation by becoming a little older.

*Hans Selye, “The Father of Stress”*

The concept of the tight interweaving of stroke with stress load is widely accepted, and the links between stress and stroke are complex and multifaceted. The association of IS with stress loads and individual stress response is confirmed by numerous studies in patients and animal models. This association is mediated by functioning of the main neurohumoral system, the HPA “stress” axis. HPA axis functions as a key system of stress response; it underlies both switching on acute stress response and mechanisms of long term adaptation. Montellano et al. (2021) have reviewed blood-based biomarkers in IS prognosis and stated that cortisol, a major stress response glucocorticoid hormone in humans, is among the most promising prognostic biomarkers of IS. Glucocorticoids (GCs) released from the adrenal cortex under the influence of stress are the most important messengers in the integrative regulation of adaptive plasticity of the brain, executed by the HPA system. Endocrine alterations of the HPA axis are among the primary stress-induced alterations after cerebral ischemia.

Ischemic stroke resulting from acute brain transient ischemic attack initiates a complex sequence of events in the central nervous system and HPA axis which may ultimately culminate in brain cell damage. The association of acute IS in humans with an increased activity in the HPA axis has been demonstrated long ago. Olsson et al. (1989) reported that IS stroke patients who underwent a dexamethasone suppression test had higher post-dexamethasone cortisol levels than control group; these levels were significantly associated with proximity of the lesion to the frontal pole of the brain. It was suggested that HPA axis activation may have a number of negative effects on organ functioning, and may be a predictor of a poorer functional outcome (Olsson, 1990). The well documented fact that hypoxia-ischemia in rodents is accompanied by increased activity of HPA axis and abundant secretion of corticosterone, the major glucocorticoid hormone in rodents, suggests that this could potentially exacerbate brain damage *via* activation of receptors of GCs and augmenting neuronal vulnerability.

The duration and extent of the HPA axis activation are regulated by numerous factors, leading to activation of HPA axis hormones: corticotropin-releasing hormone, adrenocorticotropic hormone (ACTH), cortisol (in humans), or corticosterone (in rodents). Numerous studies suggest that activation of these hormones following brain ischemia can result in neurohormonal dysfunction that can worsen long-term prognosis following stroke. Many data reported during the last half century presented evidence that changes in the HPA axis play an important role in brain ischemia (Radak et al., 2014). Fassbender et al. (1994) observed an early and persisting activation of the HPA axis in relation to IS severity. Its characteristic biphasic pattern suggests an initial central stimulation of ACTH release followed by feedback suppression associated with an increased susceptibility of the adrenal gland. Since adrenal hormones are known to exacerbate hypoxic injury to neurons, their massive release in acute stroke may increase the degree of brain damage.

An acute dysfunction of the HPA system accompanying stroke is mostly interpreted as a manifestation of acute stress. Notably, though stress is known to be one of the risk factors of stroke, few experimental or clinical studies have directly examined possible mechanisms by which stress may affect stroke outcome. In patients with pre-stroke normoglycemia, 7 p.m. cortisol on admission significantly correlated with fasting blood glucose values suggesting that hyperglycemia during the acute phase of IS is a stress response (Murros et al., 1993). Salivary cortisol was positively correlated with 24-h systolic blood pressure and night-time blood pressure, suggesting that stress is a contributing factor for high blood pressure in acute IS (Ahmed et al., 2004).

In acute IS patients, increased plasma cortisol was negatively correlated with plasma brain-derived neurotrophic factor (BDNF) levels, neurological condition, cognitive function, functional responses, and emotional status, suggesting a connection between the declines of clinical, behavioral and biochemical blood parameters with stress-induced cortisol elevation (Casas et al., 2017). Importantly, sex dependent stress reactivity may be associated with different HPA axis response to IS in males and females. Women may exhibit an attenuated cortisol response to stroke. According to the results of high-throughput profiling of the circulating proteome, there are significant sex-associated differences in GC signaling following IS (O’Connell et al., 2018). In a cohort of patients with transient ischemic attacks (TIA), serum cortisol levels were significantly increased only in females (Klimenko et al., 2016).

Measurement of cortisol in plasma and cerebrospinal fluid in acute IS patients is significant for monitoring the intensity of response of an organism to acute brain damage (Selaković, 2004). It was demonstrated that changes in HPA axis which maximize the production of cortisol occur soon after the onset of stroke. The cortisol secretion rate appeared to be a good indicator of the severity of the stress caused by IS and may be useful in predicting the prognosis of the disease (Korsić et al., 1990). Murros et al. (1993) reported that serum cortisol predicted the stroke outcome. Both the 7 a.m. and the 7 p.m. values in the initial and 1-week samples correlated positively with the severity of hemiparesis on the corresponding days. During the 3 month follow-up, the 7 p.m. values predicted better the functional outcome and case fatality. Acute IS mortality was found to be associated with increased serum cortisol levels. Christensen et al. (2004) also demonstrated an association of serum cortisol with stroke severity and markers reflecting stroke severity. Anne et al. (2007) confirmed that plasma levels of cortisol were associated with IS severity, as well as short-term functional outcomes. Zi and Shuai (2013) evaluated the prognostic value of serum cortisol in Chinese patients with an acute IS and confirmed that cortisol can serve as an independent short-term prognostic marker of functional outcome and death. The systematic review and analysis of a number of clinical IS studies suggest that stress response assessed as HPA axis activation is among the most promising prognostic biomarkers (Montellano et al., 2021). In patients with IS, cortisol was shown to be an independent prognostic marker for death and functional outcome within 90 days and 1 year, while the levels of other anterior pituitary axis hormones (peripheral thyroid hormones, growth hormone) could not reliably predict outcome in IS (Neidert et al., 2011). Cortisol and brain natriuretic peptide values predicted long-term mortality after IS, suggesting that neurohumoral disturbance associated with increase in their levels was prognostically unfavorable. Importantly, the biomarker panel (including besides cortisol also brain natriuretic peptide and copeptin, a surrogate marker for arginine vasopressin release) predicted functional outcome and death within 90 days significantly more efficiently than the National Institute of Health Stroke Scale (NIHSS) or each biomarker alone (Tu et al., 2013).

Though vast majority of groups confirmed prognostic validity of cortisol in IS, some studies did not substantiate these results. In particular, Varoglu et al. (2009) failed to substantiate that in the prediction of stroke prognosis, cortisol is a deterministic factor. Katan et al. (2011) reported that measurement of plasma cortisol levels in patients with TIA did not provide additional prognostic information beyond the ABCD2 clinical risk score. Interestingly, Olsson (1990) reported that both high and too low levels of cortisol in the blood increase the risk of death within 28 days from the onset of stroke. It is possible that any extreme dysfunction of HPA axis disturbs adaptive capability to cope with stroke.

Excessive cortisol levels after stroke were shown to be associated with delayed cognitive dysfunction. In the TABASCO prospective cohort study, individuals with higher hair cortisol concentration, which probably reflected higher long-term cortisol release, were prone to develop cognitive decline following moderate IS or TIA (Ben Assayag et al., 2017). In the same TABASCO study, associations between post-stroke cortisol levels, brain abnormalities, genetic factors, and cognitive outcome was investigated in a longitudinal stroke survivors cohort (Tene et al., 2018). Higher bedtime cortisol levels immediately post-stroke were associated with larger neurological deficits, brain atrophy, worse white matter integrity, and worse cognitive results up to 24 months post-stroke. It was suggested that high bedtime salivary cortisol levels in IS patients may provide information about dysregulation of diurnal HPA axis activity under acute challenge conditions, and predict worse cognitive outcome. These data were confirmed by Wang et al. (2021) who investigated the relationship between salivary cortisol content and secondary mild cognitive impairment (MCI). Higher salivary cortisol was associated with a higher probability of MCI occurrence and was suggested to be valid as a predictive marker for MCI. These recent studies confirm the data reported earlier by Maeda et al. (1991) who showed that HPA axis function was activated in demented patients and that this activation was related generally to dementation itself, not to the etiology of dementia.

Hypothalamic-pituitary-adrenocortical axis dysfunction is closely associated with pro-inflammatory events (Gulyaeva, 2019b). In acute IS patients, serum interleukin-6 predicted cortisol release (Szczudlik et al., 2004). The authors suggested that brain ischemia could stimulate IL (interleukin)-6 release in blood and in this way modulate HPA axis. Notably, Slowik et al. (2002) suggested that prognostic significance of hypercortisolemia in acute IS patients is related to the inflammatory response rather than to the stress response. Indeed, the relationship between GCs and inflammation still remains obscure; obviously, it is very complicated and tissue-dependent (in particular, region-dependent in the brain). After IS, patients may experience distorted systemic immunity, in particular, peripheral immunosuppression and higher susceptibility to infections, which at least in part may be attributed to lymphopenia. In patients with acute IS, increased cortisol levels inversely correlated with blood lymphocyte count suggesting that HPA axis mediates B lymphopoiesis defects after IS (Zierath et al., 2018). Increased serum cortisol after IS was independently associated with neutrophilia and lymphopenia.

Although there are quite a few clinical studies showing the involvement of HPA axis in IS stroke outcome and predictive value of cortisol (the data are summarized in the Table 1), there are still numerous mechanistic details which should be thoroughly examined. While some of the mechanisms implicating HPA axis in consequences of brain ischemia have been studied, the data remain scattered and non-systematic. The studies on animal models of IS allow to reproduce key stroke events reported in the patients and to explore deeper the corticoid-dependent mechanisms underlying IS symptoms. Global cerebral ischemia in rats induces lasting dysregulation of HPA axis function implicated in emotional and cognitive post-ischemic behavioral impairments observed in survivors of cardiac arrest and stroke (de la Tremblaye et al., 2014). Like in IS patients, ischemia modeled in rodents is accompanied by abundant secretion of corticosterone, the main rodent glucocorticoid. The earlier data that exposure of the brain to high levels of GCs during ischemia-reperfusion induced neuronal cell death were confirmed recently (Hoshi et al., 2020). In a model of phototrombotic stroke it was shown that corticosterone administration induced increased tissue loss within the ipsilateral hemisphere, and modest white matter tract reorganization affecting axons and glial cells (Zalewska et al., 2021). An 11-beta-hydroxylase inhibitor of GC synthesis metyrapone reduced rat brain damage and the incidence of seizures after hypoxia-ischemia; this effect might be only partially dependent on its effect on modulating plasma corticosterone since corticosterone administration to metyrapone-treated animals elevated plasma corticosterone but did not result in a following enhancement of brain damage (Krugers et al., 1998).

**TABLE 1:** Predictive value of cortisol in patients with IS.

Patients	Changes in cortisol/HPA axis	Predictive value/Key conclusion	References
IS patients	Acute IS patients had higher post-dexamethasone blood cortisol levels associated with proximity of the lesion to the frontal pole of the brain.	SI patients are exposed to hypercortisolism, which may have negative consequences upon organ functions.	Olsson et al., 1989
IS patients	Urinary free cortisol excretion increased in the acute phase of IS.	Acute IS is associated with increased activity in the cortisol axis. Cortisol excretion is a predictor of poorer functional outcome.	Olsson, 1990
IS patients	IS patients who died had significantly higher urinary cortisol secretion values. The first day urinary 17 oxogenic steroid excretion pointed to the greater adrenocortical response in these patients.	Cortisol secretion rate appears to be a good indicator of the severity of SI-induced stress and may be useful in predicting the prognosis of the disease.	Korsić et al., 1990
IS patients, patients with pre-stroke normoglycemia	Serum cortisol in initial and 1-week samples correlated positively with severity of hemiparesis. Acute IS mortality was associated with increasing serum cortisol levels. A correlation existed between initial 7 p.m. serum cortisol and 7 a.m. fasting blood glucose values.	Serum cortisol predicts stroke outcome during 3 month follow-up. Hyperglycemia during the acute phase of IS is a stress response	Murros et al., 1993
IS patients	Hypercortisolemia was associated with older age, greater severity of neurological deficit, larger ischemic lesions on CT, and worse prognoses. No correlation was found between serum cortisol levels and other markers of stress response. A correlation was found between serum cortisol and markers of inflammatory response.	Prognostic significance of hypercortisolemia in acute IS patients is related to the inflammatory response rather than to the stress response.	Slowik et al., 2002
IS patients, patients with transient ischemic attacks (TIA)	Significant increase of cortisol concentration in plasma and cerebrospinal fluid found in acute IS, with maximum during first 2 days. The increase was highest in patients with brain infarction and lowest in TIA patients.	Measurement of cortisol in plasma and cerebrospinal fluid in patients with acute IS is significant for monitoring intensity of response to acute brain damage.	Selaković, 2004
IS patients	Serum interleukin (IL)-6 and cortisol levels increased in acute IS. In IS patients, IL-6 level correlated significantly with cortisol level; morning serum IL-6 level independently predicted evening/night cortisol level.	Brain ischemia stimulates IL-6 release in blood and in this way modulates HPA axis.	Szczudlik et al., 2004
IS patients	Salivary cortisol positively correlated with 24-h systolic blood pressure and night-time blood pressure in acute IS patients.	Stress is a contributing factor for high blood pressure in acute IS.	Ahmed et al., 2004
IS patients	Serum cortisol in acute IS was independently related to death within 7 days of stroke onset, but was not a predictor of death or dependency within 3 months. Serum cortisol correlated with brain lesion volume.	Serum cortisol levels are associated with stroke severity and other markers reflecting stroke severity.	Christensen et al., 2004
IS patients	Higher levels of plasma cortisol and adrenocorticotropic hormone (ACTH) were observed in IS patients who died during the follow-up. Cortisol levels were associated with catecholamine levels and measures of neurologic deficit.	Plasma levels of cortisol are associated with IS severity and short-term functional outcomes. High acute phase cortisol levels predicts long-term mortality after SI.	Anne et al., 2007
IS patients	Cortisol levels at 3rd and 7th days after SI correlated with lesion volumes, but in prediction of IS prognosis, cortisol was not a deterministic factor.	The study failed to substantiate significance of blood cortisol in the prediction of stroke prognosis.	Varoglu et al., 2009
TIA patients	The study assessed prognostic reliability of 2 distinct stress hormones, copeptin and cortisol, for the risk stratification of re-events in patients with TIA.	In patients with TIA plasma cortisol does not provide additional prognostic information beyond the ABCD2 clinical risk score.	Katan et al., 2011
IS patients	Cortisol was an independent prognostic marker of functional outcome and death in patients with IS within 90 days and 1 year.	Though cortisol is an independent prognostic marker for death and functional outcome in SI, it adds no significant additional predictive value to National Institutes of Health Stroke Scale (NIHSS) score.	Neidert et al., 2011
IS patients	Plasma levels of cortisol and copeptin were associated with stroke severity and short-term functional outcomes and remained independent outcome predictors after adjusting for all other significant outcome predictors.	Plasma cortisol predicts long-term mortality after IS. Increase of cortisol level in blood is prognostically unfavorable.	Tu et al., 2013
Chinese patients with acute IS	Positive correlations were found between levels of cortisol and NIHSS score, glucose levels and infarct volume. Cortisol was an independent prognostic marker of functional outcome and death even after correcting confounding factors.	Cortisol is an independent short-term prognostic marker of functional outcome and death. Cortisol can improve the NIHSS score in predicting short-term functional outcome.	Zi and Shuai, 2013
TIA patients	Serum cortisol levels were significantly increased only in females with TIA.	Cortisol response in TIA is sex-dependent.	Klimenko et al., 2016
IS patients	In acute IS patients, increased plasma cortisol negatively correlated with plasma brain-derived neurotrophic factor (BDNF), neurological condition, cognitive function, functional responses, and emotional status.	Declines in clinical, behavioral, and biochemical blood parameters are associated with stress-induced cortisol elevation.	Casas et al., 2017
IS patients	In patients with acute IS, increased cortisol levels inversely correlated with blood lymphocyte count. After IS, increased serum cortisol was independently associated with neutrophilia and lymphopenia.	HPA axis mediates B lymphopoiesis defects after IS.	Zierath et al., 2018
IS patients, TIA patients	Higher hair cortisol concentration were significantly associated with a larger lesion volume and worse cognitive results 6, 12, and 24 months post-stroke. Higher hair cortisol at baseline was significant risk factor for cognitive decline, after adjustment for age, gender, body mass index, and apolipoprotein E4 (APOE E4) carrier status.	Individuals with higher hair cortisol concentration are prone to develop cognitive decline following moderate IS or TIA.	Ben Assayag et al., 2017
Longitudinal IS survivors cohort	Higher bedtime salivary cortisol levels immediately post-stroke are associated with larger neurological deficits, brain atrophy, worse white matter integrity, and worse cognitive results up to 24 months post-stroke.	High bedtime salivary cortisol post-stroke provides information about dysregulation of diurnal HPA axis activity and predicts worse cognitive outcome.	Tene et al., 2018
Mild cognitive impairment (MCI) after IS	Higher salivary cortisol was associated with a higher probability of secondary MCI occurrence after IS.	Salivary cortisol level is an independent risk factor of MCI after IS and can be used as a predictive marker for MCI.	Wang et al., 2021
IS patients	Systematic review of clinical SI studies	Stress response (cortisol levels, HPA axis activation) is among most promising prognostic biomarkers.	Montellano et al., 2021

It was shown in a rat middle cerebral artery occlusion (MCAO) model of IS that acute stress could increase brain ischemic damage, the effect of stress being mediated at least partially by pro-inflammatory cytokines IL-1β and tumor necrosis factor (TNF)-α (Caso et al., 2006, 2007). The functioning of HPA system is associated with neuroinflammation, the development of which in stroke is associated with increased expression of pro-inflammatory cytokines TNF-α, IL-1β, and IL-6. These cytokines can modulate the size of ischemic damage in experimental IS in rodents (Davies et al., 1999), and their level increases in the cerebrospinal fluid and blood after IS in humans (Simi et al., 2007; McCoy and Tansey, 2008; Whiteley et al., 2009). All three pro-inflammatory cytokines, and especially IL-1β, are able to activate HPA axis and cause an increase in the level of corticosterone in the systemic bloodstream (Dunn, 2000; John and Buckingham, 2003).

Similarly to IS patients, transient MCAO in mice induced GC release associated with B lymphopoiesis defects (Courties et al., 2019). In the experiments with cell type-specific deletion or overexpression of glucocorticoid receptor (GR) in mice with ischemia, GR signaling in myeloid cells increased Iba-1 (ionized calcium binding adaptor molecule 1) and CD68 (cluster of differentiation 68 protein) staining as well as nuclear p65 (component of nuclear factor kappa-light-chain-enhancer of activated B cells) levels in the injured tissue. GCs also reduced levels of occludin, claudin 5, and caveolin 1, proteins essential to blood-brain-barrier integrity; these effects required GR in endothelial cells (Sorrells et al., 2013). Notably, GCs compromised neuron survival, an effect mediated by GR in myeloid and endothelial cells to a greater extent than by neuronal GR.

Glucocorticoids have essential effects on glucose metabolism in both IS patients and IS models and the glucose paradox of brain ischemia (aggravation of delayed ischemic neuronal damage by hyperglycemia) may be closely related to GCs. Glucose load was shown to induce a brief increase in GCs release. When brain ischemia in the rat was accompanied by glucose-induced elevated levels of corticosterone, an aggravation of the ischemic outcome was demonstrated, while both the blockade of corticosterone increase by “chemical adrenalectomy” with metyrapone or the blockade of GR in the brain with mifepristone (RU486) prevented the exacerbating effect of pre-ischemic hyperglycemia on post-ischemic outcome (Schurr, 2002; Payne et al., 2003). Since delayed neuronal damage induced by pre-ischemic hyperglycemia correlated with corticosterone rather than with glucose levels, it was suggested that corticosterone may have a greater prognostic value than glucose in predicting cerebral ischemic damage.

## Glucocorticoid-Mediated Remote Hippocampal Damage as Potential Mechanism of Delayed Consequences of Ischemic Stroke

The best material model of a cat is another, or preferably the same, cat.

*Arturo Rosenblueth, as co-author with Norbert Wiener, in ‘The Role of Models in Science’, Philosophy of Science (1945), 12, 320.*

Thus, both clinical data and the results from rodent models of IS show that GCs released during the activation of the HPA axis are involved in stroke-induced brain dysfunction. Recently, the data have been accumulating, indicating that the damage after a stroke is not limited to the infarction area only, but also spreads to non-ischemized regions of the brain, causing secondary, frequently, remote and delayed, damage. For example, after focal ischemic damage to the cerebral cortex and/or striatum, secondary changes are observed in areas of the brain remote from the infarction zone, primarily in the hippocampus (Butler et al., 2002; Block et al., 2005).

Although brain damage caused by IS primarily affects the cerebral cortex, it has been hypothesized that essential mechanisms of IS-induced cognitive and depressive disorders are associated largely with the hippocampus (Gulyaeva, 2019a). The adrenal stress hormones GCs acting through their receptors abundant in the hippocampus are critical to physiological control of different executive functions and behavioral responses which usually represent adaptive reactions. Signal transduction mediated by hippocampal receptors of GCs regulate genomic activities underlying neuroplasticity and behavioral adjustment to stressogenic factors (Gulyaeva, 2019b). Milot and Plamondon (2011) demonstrated increased sensitization and responsiveness of HPA system at long intervals after cerebral ischemia in rodents. These effects contributed to post-ischemic cognitive impairments (disturbances in spatial memory) and hippocampal degeneration.

Kadar et al. (1998) reviewed numerous earlier studies, using five different experimental models in rats (normal aging, hypoxia, prolonged corticosterone administration, brain ischemia, and cholinesterase inhibition), showing that cognitive dysfunction is invariably accompanied by hippocampal CA1 and CA3 pyramidal cells degeneration, though the most affected area depended on specific model used. Rami et al. (1998) focused on synergy between chronic corticosterone treatment and cerebral ischemia in producing damage to hippocampal neurons. Their data support the hypothesis that corticosterone treatment accompanied by an ischemic insult, cause an extended increase in neuronal [Ca2+], non-calbindinergic neurons being particularly prone to ischemic insults.

More than 3 decades ago, Sapolsky and his group suggested that GCs disrupt the energy metabolism of neurons of the hippocampus and enhance glutamatergic/NMDA signals making neurons more vulnerable to metabolic insults. This suggestion was confirmed by the data of *in vitro* (Sapolsky et al., 1988) and *in vivo* experiments (Armanini et al., 1990). Excitatory synapses are considered as key elements in synaptic plasticity and behavioral adaptation and their dysfunction is involved in cognitive and emotional disturbances as well as in epilepsy. At present, numerous other mechanisms of GC control of the glutamatergic synapse have been discovered supporting IS-induced GC-mediated hippocampal dysfunction. GCs, by triggering signal transduction through mineralocorticoid receptors and GRs located on synaptic membranes and in the cytosol of glutamatergic neurons of the hippocampus, regulate synapse plasticity at the level of pre- and post-synaptic compartments (see Gulyaeva, 2021c for review). GCs modulate synapse excitability through changes in vesicular transport and glutamate release, and mediate changes in the expression, composition, and properties of ionotropic NMDA and AMPA receptors, as well as other glutamate receptors. Since hyperglutamatergic transmission is regarded as key mechanism of hippocampus-dependent cognitive, affective, and epilepsy-associated disturbances, it is more than probable that it is one of the main players in the delayed consequences of IS (Gulyaeva, 2021b).

Both in rats and in gerbils, adrenalectomy protected hippocampal pyramidal cells from transient ischemia (Morse and Davis, 1990). These findings suggest that GCs affect the rate of hippocampal pyramidal cell disappearance following ischemia. Metyrapone reduced brain injury induced by focal and global ischemia. Experiments with metyrapone demonstrated that in rodent models of ischemia, the GC-mediated stress-response exacerbated consequent hippocampal damage (Smith-Swintosky et al., 1996). Metyrapone prevented hypoxia/ischemia-induced loss of synaptic function in the rat hippocampus, while corticosterone treatment worsened consequences of ischemia (Krugers et al., 2000). It has been concluded that preventing ischemia-induced increase in GC levels rather than blocking the GR preserves both synaptic function and cellular integrity of the hippocampus. It has been noticed above that cytokines, critical mediators of local and systemic inflammatory responses, are produced in the brain following IS. Using a MCAO model of IS, Onufriev et al. (2017) showed that accumulation of corticosterone in the hippocampus was associated with an increase of proinflammatory cytokine IL-1β.

Dementia that occurs after a stroke demonstrates a high (up to 90%) comorbidity with depression (Whyte et al., 2004). Depression after an IS is diagnosed in 40–70% of patients, depending on a cohort studied (Hachinski, 1999), incidence of post-stroke depression ranging from 11 to 41% within 2 years (Guo et al., 2021). As mentioned above, activation of HPA system is one of the first physiological responses to cerebral ischemia, it occurs in the first hours after ischemia and leads to a prolonged increase in the concentration of GCs in the blood (Fassbender et al., 1994; Johansson et al., 1997; Marklund et al., 2004). It is generally assumed that the dysregulation of HPA axis and pre-disposition to stress-induced mental diseases, including depression, are based on a violation of the GCs regulation of the feedback system, as well as an imbalance between the central corticosteroid receptors (Sapolsky, 2000; Robertson et al., 2005; Sarabdjitsingh et al., 2009). The modulation of HPA axis activity is implemented, particularly, by the hippocampus (Jacobson and Sapolsky, 1991), through corticosteroid receptors which are highly expressed in the hippocampal fields CA1, CA2, CA3 and the dentate gyrus (Sarabdjitsingh et al., 2009). It is known that the hippocampus is an inhomogeneous structure, displaying septo-temporal (dorso-ventral) gradient. The dorsal and ventral parts of the hippocampus execute different functions, with the former mainly associated with cognition, and the latter with stress reactions and emotions (Segal et al., 2010; Gulyaeva, 2019a). Thus, the hippocampus controls both cognitive and emotional functions. The above reasons brought about a new hypothesis on the distant hippocampal damage as a key link in the pathogenesis of cognitive and psychiatric disturbances after focal brain injury, including delayed consequences of IS. According to this hypothesis, excess of GCs secreted after a focal brain damage (e.g., IS), in particular in patients with abnormal stress-response due to HPA axis dysfunction, interacts with receptors of GCs in the hippocampus inducing signaling pathways which stimulate neuroinflammation and subsequent events including disturbances in neurogenesis and hippocampal neurodegeneration (Gulyaeva, 2019b). Thus, functional and structural damage to the hippocampus, a brain region selectively vulnerable to external factors and responding to them by increased cytokine secretion, may form the basis for cognitive function disturbances and psychopathology development.

Besides cognitive and emotional post-stroke disturbances, stroke may trigger epileptogenesis, often comorbid with depression. Common molecular and cellular mechanisms of these comorbidities include distant hippocampal damage associated with HPA axis dysfunction, the malfunction of GR, development of neuroinflammation, leading to neurodegeneration and loss of hippocampal neurons, as well as formation of aberrant neural networks (Gulyaeva, 2021a). GC-mediated mechanisms of hippocampal damage include alterations in subgranular neurogenesis, these disturbances contributing to both cognitive and emotional disturbances and to epileptogenesis as well (Podgorny and Gulyaeva, 2021).

It should be noted that though the above mentioned mechanisms of hippocampal damage were revealed and confirmed in animal models of brain ischemia, at present there are not enough clinical data to unequivocally show this causal relationship of respective mechanisms and events in clinical studies, including patients after IS. Thus, the hypothesis of IS-dependent remote and delayed hippocampal damage can be finally confirmed or refuted after relevant clinical data will be available.

## Discussion: Appropriate Models and Extensive Mechanistic Studies Wanted

A theory has only the alternative of being right or wrong. A model has a third possibility: it may be right, but irrelevant.

*Manfred Eigen, ‘The Origin of Biological Information’, in Jagdish Mehra (ed.), The Physicists’s Conception of Nature (1973), 618.*

Though increased cortisol in IS is regarded as a biomarker of higher mortality and worse recovery prognosis, the detailed mechanisms of HPA axis dysfunction involvement in delayed post-stroke hippocampus-associated cognitive and emotional disorders remain obscure. This point is of great translational importance: if HPA axis disturbances are related to deferred post-stroke pathologies, it should be considered in the elaboration of advanced stroke therapies.

One of the key question and obvious growing point is the influence of stress hormones GCs on the brain, and, specifically, hippocampus, in different periods after IS. Association of GCs with neuroinflammation is one of the critical issues. GCs are well known for being anti-inflammatory, but some reports suggest that GCs can also augment some aspects of inflammation during acute brain injury thus opposing classical anti-inflammatory actions of GCs. Onufriev et al. (2017) demonstrated accumulation of cortisol and IL-1β in the hippocampus of rats during the acute period after MCAO, suggesting the association of GC excess with neuroinflammation. However, some earlier studies demonstrated protective effects of GCs in brain ischemia. Prolonged corticosterone treatment resulted in down-regulation of the HPA axis and lower plasma corticosterone levels during hypoxia/ischemia in rats and a reduction in subsequent brain damage (Krugers et al., 1995). Tuor and Del Bigio (1996) and Tuor (1997) reported about protection against hypoxic-ischemic damage in neonatal rats with corticosterone and dexamethasone, while glucocorticoid antagonist RU38486 inhibited the beneficial effects of dexamethasone suggesting that dexamethasone-dependent neuroprotection was mediated by GR. The ambiguity of understanding the involvement of cortisol in IS to a certain extent reflects the ambiguity of the pro - and anti-inflammatory effects of GCs. Bolshakov et al. (2021) analyzed the results of studies on pro- and anti-inflammatory effects of GCs in the hippocampus in different models of stress and stress-related pathologies. The available data form a sophisticated, though often quite phenomenological picture of a modulatory role of GCs in hippocampal neuroinflammation. Understanding the dual nature of GC-mediated effects as well as causes and mechanisms of switching between them can provide us with effective approaches and tools to avert hippocampal neuroinflammatory events and as a result to prevent and treat brain diseases developing after IS.

In this review, the current state of clinical and experimental research on the potential involvement of the HPA axis in delayed post-stroke disorders was analyzed with particular focus on remote GC-induced hippocampal damage and its contribution to the development of neurological and emotional disorders. IS models used in pre-clinical studies should take into account the state of HPA axis for accurate interpretation of the data, in particular long term effects of drugs. At present, although the involvement of augmented cortisol in IS outcome and delayed post-stroke brain disturbances is recognized, this knowledge and respective hypotheses did not affect the pre-clinical studies as well as clinical treatment.

Hypothalamic-pituitary-adrenocortical axis response may critically depend on the model of IS used in the pre-clinical study. Recently we have shown that both corticosterone accumulation in the hippocampus, and the pro-inflammatory trend may be dependent on the specific details of the surgery in the MCAO model, one of most popular models of IS used in pre-clinical studies (Kasatkina et al., 2021). Two classical surgical approaches for intraluminal filament MCAO, Longa and Koizumi methods, which are exploited as alternatives, have been compared. A direct comparison of these models in mice (Smith et al., 2015; Morris et al., 2016) showed both similarities and critical differences between them. In our study, a comparison of these two models in rats have been performed (Kasatkina et al., 2021). Though neither infarct volume at day 3, nor neurological deficit and weight loss differed in these models, the mortality rate tended to be higher in the Koizumi model group. While both groups showed an increase of ACTH levels in blood serum 3 days after the surgery, only rats in the Koizumi MCAO model group demonstrated an increase in blood corticosterone and IL-1β versus respective sham groups. Corticosterone accumulation was detected in the ipsilateral frontal cortex and hippocampus of both groups, and in the contralateral hippocampus of the Koizumi model group. IL-1β levels increased in the ipsilateral hippocampus and bilaterally in the frontal cortex of the Koizumi MCAO model group, but not the Longa model group. The data demonstrate differences in two MCAO models and suggest that Koizumi model of MCAO pre-disposes to corticosterone-dependent distant neuroinflammatory hippocampal damage. This is an additional example of potential differences in the MCAO models, equally used in pre-clinical studies to induce stroke in rodents, though it is obvious that they imitate different clinical cases. The dissimilarities between these two models should be taken into account in the interpretation, comparison, and translation of pre-clinical results.

## Conclusion

One of the growth points in bridging the gap between IS theory and clinic is to consider the involvement of HPA activation and of the hippocampal dysfunction at different stages of IS. It is vital to study distant hippocampal (“secondary”) damage and its delayed consequences in pre-clinical and clinical research. Models of IS with and without HPA axis dysfunction should be validated. We need models that reproduce the state of the HPA axis in clinical cases, and this should be considered when planning the pre-clinical research. In clinical studies of IS, it is useful to re-evaluate diagnostic and prognostic potential of cortisol and other HPA axis hormones. Finally, it is important to reveal IS patients with permanently disturbed HPA axis. Patients-at-risk with high cortisol and potential delayed remote hippocampal damage should be monitored since potential hippocampal dysfunction may be the basis for development of post-stroke cognitive and emotional disturbances, as well as epilepsy.

## Author Contributions

NG: conception of the work, data analysis and interpretation, and critical revision of the manuscript. MO and YM: data collection and drafting the manuscript. NG, MO, and YM: final approval of the version to be published. All authors contributed to the article and approved the submitted version.

## Conflict of Interest

The authors declare that the research was conducted in the absence of any commercial or financial relationships that could be construed as a potential conflict of interest.

## Publisher’s Note

All claims expressed in this article are solely those of the authors and do not necessarily represent those of their affiliated organizations, or those of the publisher, the editors and the reviewers. Any product that may be evaluated in this article, or claim that may be made by its manufacturer, is not guaranteed or endorsed by the publisher.
